# Chalk stream restoration: Physical and ecological responses to gravel augmentation

**DOI:** 10.1371/journal.pone.0313876

**Published:** 2024-11-20

**Authors:** Lewis A. Dolman, Andrew S. Vowles, Paul S. Kemp

**Affiliations:** International Centre for Ecohydraulics Research, Faculty of Engineering and Physical Sciences, University of Southampton, Hampshire, Southampton, United Kingdom; Universitat de Barcelona, SPAIN

## Abstract

To mitigate the morphological and ecological impacts of direct (e.g. dredging) and indirect (e.g. damaged river function) sediment loss, gravel augmentation is commonly practiced in river systems globally. Despite this, the effectiveness of this practice remains poorly understood, especially in less often considered systems such as chalk streams which present uncommon conditions (e.g. low stream power, stable flow) and may respond to interventions in ways that differ from systems more commonly studied. This study quantified immediate (0–1 years) and short-term (1–2 years) physical and ecological responses to gravel augmentation at two English chalk stream restoration sites: Home Stream (HS; River Test) and East Lodge (EL; River Itchen). We quantified habitat (depth, velocity, substrate composition), cover of different macrophytes, and macroinvertebrate (before-after-control-impact) abundance and community structure. Restoration reduced depth and increased gravel cover in both sites and decreased the cover of filamentous green algae in HS. Macroinvertebrate communities became more dominated by silt-intolerant taxa, while abundance [HS only] and taxon richness increased 1–2 years post-restoration. Whilst the responses found were generally positive in light of the restoration goals, the effects varied across sites, post-restoration time periods and ecological groups, emphasising the need for the more holistic monitoring of restoration projects considering community-level responses at different sites and systems over ecologically relevant timescales. This will help inform on the generality and longevity of responses and provide the evidence needed to develop sound restoration practice.

## 1. Introduction

For centuries, humans have intensively modified rivers for agriculture, navigation, flood defence, industrial and domestic water supply and energy generation [1, 2]. As a consequence, rivers have been dammed, channelised, dredged, and cut-off from their flood plains, disrupting natural hydrogeomorphological regimes [3, 4], reducing fluvial connectivity [5] and homogenising habitat [6]. Extensive river modification has reduced biodiversity [7], modified communities of fauna and flora [8] and degraded ecological quality [9], contributing to global threats to fresh waters and associated potential for humanitarian crisis [10].

Localised depletion of river sediments through direct (e.g. dredging and mining [11, 12]) and indirect (e.g. interruption of sediment regime by dams [13]) mechanisms threatens morphological and ecological fluvial integrity [14, 15]. Morphologically, sediment depletion can promote channel incision, bank instability and armouring of the river bed [14, 15], reduce habitat heterogeneity [16] and inhibit channel dynamics [17, 18]. Ecologically, it can negatively impact biodiversity, communities and food web dynamics [11, 19], habitat suitability for substrate-spawning taxa [20, 21], and promote the establishment of non-native species [19]. Globally, efforts have increased to mitigate the negative impacts associated with sediment depletion through rehabilitation measures, including gravel augmentation, one of the more common substrate restoration techniques [22–24].

The artificial addition of gravels to degraded rivers has been widely employed in several regions, with examples from North America (United States [25], Canada [26]), Europe (United Kingdom [27], Germany [28], Norway [29]) and Asia (Japan [30]). This practice can help facilitate the regeneration of natural processes and be used to restore riverbed structure (e.g. by redistributing silt) and features over a range of scales [24]. Those operating at the reach-scale tend to focus on re-naturalising riverbed structure and features, e.g. to create riffles [31] and gravel bars [32], and improve ecological utility, e.g. salmonid spawning habitat quality [33]. At larger scales, efforts are directed at re-establishing dynamic processes, e.g. the formation and evolution of geomorphic features [34]. However, logistical constraints (e.g. land ownership) and the costs of implementation and monitoring can result in bias (e.g. in publication [29, 35]) towards the more common reach-scale gravel augmentation projects [36].

Despite the widespread use of gravel augmentation to help restore rivers [23], the ecological responses observed are often variable and remain poorly understood [35]. On one hand, the addition of gravel can enhance fish habitat, particularly for gravel spawning taxa [29, 33, 35, 37], macroinvertebrate diversity [38] and density [32, 35], and macrophyte species richness [39]. On the other, several studies have indicated limited (e.g. fish [31], macroinvertebrates [40, 41]), temporary (e.g. fish [28], vegetation [42]), mixed (e.g. fish [43]) and negative effects (e.g. reduced macroinvertebrate abundance and biomass [44], reduced macrophyte diversity, biomass and cover [35]). A lack of understanding of the mechanisms that underpin the ecological responses observed may be attributed to multiple factors, including limited monitoring [45], insufficient time-scales of appraisal [46], and the potential for catchment scale processes to overshadow reach scale outcomes limiting or resulting in short-term responses [31, 47]. Moreover, the general focus on single ecological indicators, especially salmonids [29], and the bias to certain regions and river types [23, 32, 44, 48] may constrain understanding of the value of gravel augmentation as an effective restoration strategy.

The majority of groundwater fed chalk streams are found in southern and eastern England, and typically exhibit low power and relatively stable flow and temperature regimes [49]. Chalk streams have been highly modified over many centuries (e.g. for agriculture, mechanical power and flood water conveyance [50, 51]), including dredging and impoundment, which has contributed to their widespread degradation [52]. Indeed, 85% of English chalk streams failed to achieve good ecological status under the European Union Water Framework Directive (2000/60/EC) in 2019, with physical modification contributing to 35% of these failures [53, 54]. Physical habitat degradation, such as that due to infrastructure, alongside intensive water abstraction and land use practices, have widely disrupted natural sediment regimes of chalk streams [51, 55]. Due to their stable hydrogeomorphology and naturally low levels of sediment recruitment and bedload mobilisation [56, 57], alongside impoverished base flows and highly modified channels, the natural regeneration of sediment is difficult without appropriate intervention [52]. Consequently, the replenishment of gravels has become an important management strategy in the restoration of chalk streams [58]. However, few studies have evaluated the ecological consequences of gravel augmentation in these systems, and those that have tend to focus on single ecological indicators (e.g. macroinvertebrates [27]).

This study quantified physical and ecological responses to gravel augmentation at two case study chalk stream restoration sites in southern England. Restoration was predicted to have: (1) an immediate effect on physical habitat and that this would be maintained throughout the duration of the study period (two years post restoration). Specifically, when compared with the conditions prior to restoration and at a control site, the restored sections were expected to be characterised by greater velocity and depth variability, higher maximum velocities and shallower water depth, and higher coarse-substrate cover. These physical responses were predicted to result in: (2) changes to the stream ecology, considered in terms of enhanced richness, abundance and/ or cover of aquatic fauna and flora, and shifts in community composition towards those considered characteristic of chalk stream environments (e.g. greater presence of Ephemeroptera, Plecoptera and Trichoptera [EPT] and rheophilic and silt-intolerant taxa such as *Ranunculus* spp. [50, 52]). Finally, it was expected that: (3) time would play an influential role in the ecological response, with limited or potentially negative changes associated with the immediate disturbance of habitat in the year following restoration (especially for benthic groups), followed by a shift to the aforementioned outcomes thereafter. To test these predictions, this study focused on two ecological indicators: (a) macrophytes (total macrophyte cover and cover of different macrophyte types); and (b) macroinvertebrates (abundance, taxon richness, percentage of abundance [EPTA] and taxon richness [EPTN] of the community comprised of EPT, Proportion of Sediment-sensitive Invertebrates [PSI] [59] and Lotic Index for Flow Evaluation [LIFE] [60]). To investigate the influence of time, physical and ecological responses were monitored throughout the year immediately after (immediate response) and between one and two years (short-term) post restoration.

## 2. Materials and methods

### 2.1 Study sites

**Fig 1:**
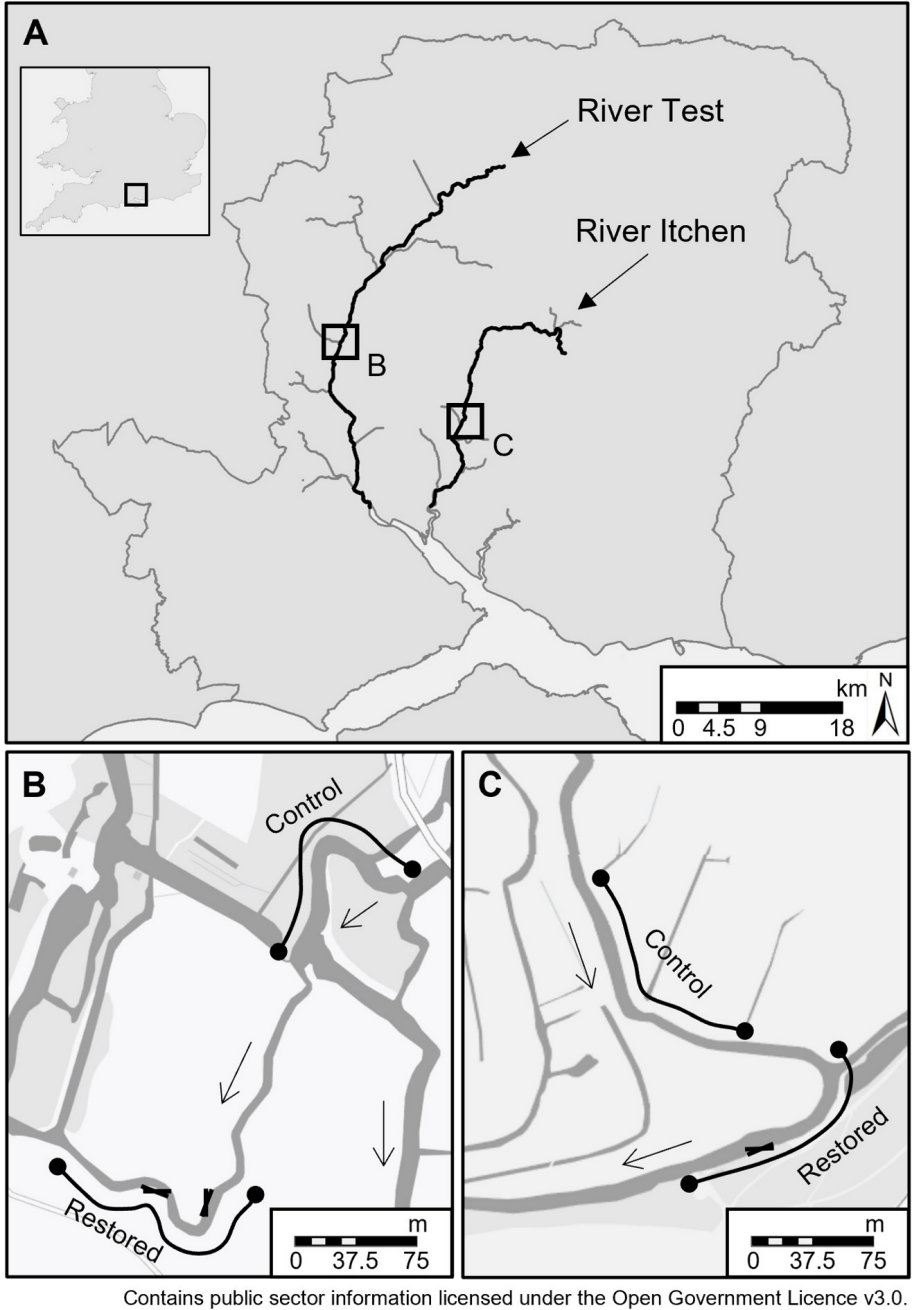
(A) Location of study sites on the Rivers Test and Itchen in Hampshire, southern England, United Kingdom, with greater detail provided for (B) Home Stream (HS) 51.07296, -1.51685, and (C) East Lodge (EL) 51.00047, -1.32551. The arrows, black lines and crosses respectively indicate flow direction, extent of control and restored reaches and positions where felled trees were secured. Country and county shapefiles and river network were supplied from [68, 69], respectively. Detailed maps (B and C) were supplied from [70].

This study monitored the physical and ecological response of two gravel augmentation restoration projects on the Rivers Test (Home Stream [HS]) and Itchen (East Lodge [EL]; Fig 1), Hampshire, United Kingdom. Both rivers (catchment area: 1,260 km^2^ and 470 km^2^, respectively) rise in the north of the county and flow southwards to join Southampton Water. In the upper catchment the geology is dominated by chalk, transitioning to sands and clays in the lower reaches [61]. Land use is predominantly arable and pasture, with dispersed settlements in the upper catchment and becoming increasingly urbanised as the rivers flow downstream [61]. Both rivers contribute to the local economy, including through recreational fly fishing and aquaculture [62]. They have also been designated as Sites of Special Scientific Interest, a conservation designation under the United Kingdom’s Wildlife and Countryside Act (1981) which protects the best wildlife and geological sites [63], for distinct habitats (e.g. fen meadows) and species (e.g. Southern damselfly [*Coenagrion mercuriale*]; [64, 65]). The River Itchen receives additional protection as a Special Area of Conservation under Directive 92/43/EEC on the Conservation of Natural Habitats and of Wild Fauna and Flora (i.e. Habitats Directive) [66], due to its faunal (e.g. European bullhead [*Cottus gobio*]) and floral (e.g. *Ranunculus fluitantis*) communities [67].

Both the River Test and Itchen have been heavily modified over many centuries. Reasons for this include the generation of mechanical power, navigation, and agriculture [62]. As a result the rivers host a high density of low-head weirs and leats and many reaches are heavily channelised, unnaturally incised and widened, and characterised by low uniform velocity regimes and fine sediment deposition [62, 71]. Additionally, catchment scale land-use has resulted in high diffuse pollutant input and associated nutrient enrichment [72], fine sediment run-off [56, 73] and over abstraction [74]. Ecologically, both rivers are considered highly degraded and suffering from “Chalk Stream Malaise”, a deterioration from the classic chalk stream habitat [75]. Indeed, in 2019, 35% of the River Test and 14% of the River Itchen Water Framework Directive monitoring sites were considered heavily modified (physically altered by human activity and substantially changed in character [76]), and 52% and 50% of sites failed to achieve ‘good ecological status’, respectively [54]. Physical modification of the channel was the primary driver of these failures (Test = 75%, Itchen = 42% [53]).

At each study site, a reach of river approximately 200 m in length was restored in October 2019 with the goal of increasing in-river habitat heterogeneity and enhancing biodiversity and conditions for *Ranunculus* spp. and gravel spawning brown trout and Atlantic salmon (*Salmo salar*). Approximately 1500 and 3000 tonnes of washed gravel substrates were deposited along the HS (natural gravel excavated from a “borrow pit” on site > 10 m away from the river to reduce costs and ensure suitability of substrate; predominantly 2–64 mm but with some finer and coarser grain sizes) and EL (predominantly 16–30 mm imported washed river gravel) reaches, respectively. This was intended to reduce depth, remobilise silt, reprofile the planform, recover a more naturalised riverbed and create geomorphic features. Localised cobble sized substrate was also placed at EL. Additionally, at each site, a limited number of felled trees (approximately 2) were secured at the riverbank to form low velocity, sheltered habitats and *Ranunculus* spp. were sparsely ‘seeded’ (approximately 1 plant per 5 m^2^) throughout the reaches via translocation from unrestored areas nearby.

A single restored reach paired with a control (both 150 m in length) were monitored at both restoration sites (Fig 1). Control reaches were selected based on their proximity and similarity to the restored sites prior to the rehabilitation work. As such, we expected restored and control reaches to be similar prior to restoration, but that the restored reach would change according to the aforementioned predictions following restoration. To prevent downstream effects of the interventions the control sites were located 200 m and 150 m upstream of the restored sections at HS and EL, respectively. At HS, the only suitable control was located upstream of a 1.1 m high weir. A before-after-control-impact (BACI) study design was used to quantify changes in physical habitat, macrophytes and macroinvertebrates. Access permission was granted by the relevant property manager prior to each data collection period.

### 2.2 Physical habitat

**Fig 2:**
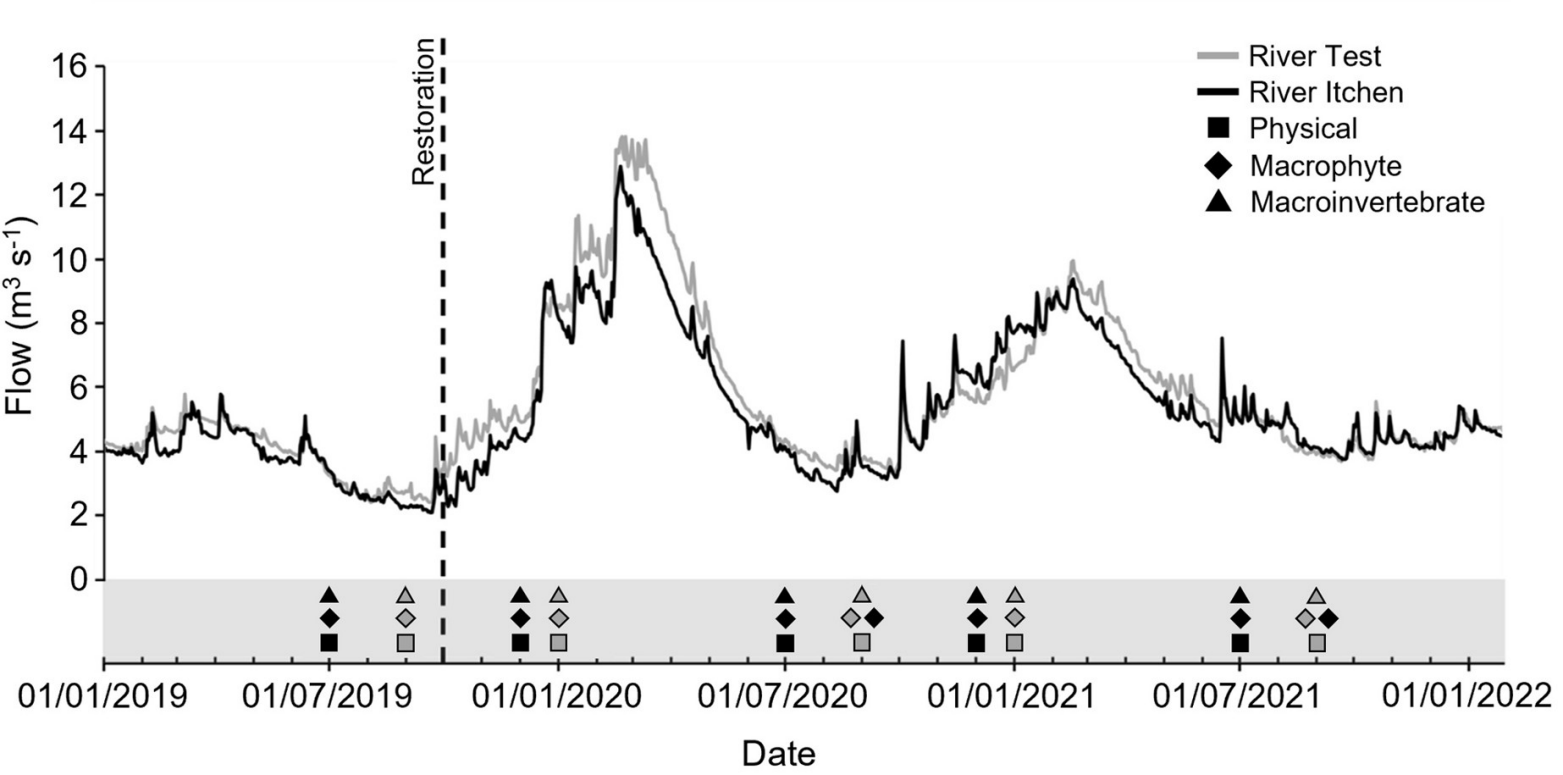
River discharge (m^3^ s^-1^) for the Rivers Test (HS) and Itchen (EL) during a study to quantify physical and ecological response to gravel augmentation. Symbols in grey shaded area show the months during which data was collected at HS (River Test, grey symbols) and EL (River Itchen, black symbols). Flow data was obtained from Chilbolton (River Test) and Highbridge (River Itchen) gauging stations, approximately 10 km upstream and 2 km downstream of the restored sites, respectively [77]. Contains public sector information licensed under the Open Government Licence v3.0.

Physical habitat was measured at five equidistant points across 16 transects located at 10 m intervals during five surveys per restoration project (see Fig 2 for a summary of data collection periods and the flow conditions under which surveys were conducted). For EL, physical data were collected during July 2019 [pre-restoration], December 2019 and July 2020 [immediate response] and December 2020 and July 2021 [short-term response]. For HS, data were collected during September 2019 [pre-restoration], January and September 2020 [immediate response] and January and September 2021 [short-term response]. At each point, depth (cm), velocity (m s^-1^; average taken over 10 secs at 1 Hz and 60% depth using a Valeport Model 801 flow meter; it was not possible to measure velocity at one point in the EL restored reach in December 2019) and the dominant surface substrate (silt [0.0039–0.123 mm], sand [0.125–2 mm], gravel [2–64 mm], cobble [> 64 mm]) within a 0.5 m^2^ quadrat was recorded. Wetted widths (m) were measured at each transect, although for some sampling periods and transects this was not possible due to excessive riparian growth and safety concerns. Depth (DCSV) and velocity (VCSV) cross sectional variability, a measure of habitat heterogeneity, was calculated by taking the standard deviation of the depth and velocity measurements taken across each transect.

### 2.3 Macrophytes

Total macrophyte cover and submerged macrophyte group cover were quantified on five and seven occasions at HS and EL, respectively. Surveys took place at the same time as the physical data collection, with the exception that September 2020 [immediate response] and 2021 [short-term response] were opportunistically also surveyed in EL. Using the same transects as for the physical habitat metrics, total macrophyte cover was assessed by estimating the percentage cover (including emergent and submerged plants) within a 0.5 m^2^ quadrat at each point (five per transect). Additionally, at the same transects, the cover of five submerged macrophyte groups (Table 1) was calculated (eight transects were conducted at 20 m intervals in the EL restored reach due to time constraints during the first data collection period). The overall macrophyte cover within a prescribed area (1 m length spanning the width of the river) was estimated using the mean of the five total macrophyte cover and wetted width measurements at each transect. The amount of overall cover comprising each macrophyte group was estimated for each transect (S1 Appendix for example of calculation). To control for limited accuracy of estimates of group cover in HS when turbidity was high, further analysis was restricted to data obtained during September when turbidity was low. Where wetted widths were not recorded (EL September 2020 and 2021, HS control September 2019), widths measured during the other sampling periods under similar flow conditions were used.

**Table 1:** Groups of submerged macrophytes recorded across transects to monitor ecological change following gravel augmentation as part of chalk stream restoration projects.

Group	Description	Typical species
Water crowfoot	Any species from the *Ranunculus* genus.	*Ranunculus penicillatus ssp*. *pseudofluitans*
		*Ranunculus aquatilis*
Water starwort	Any species from the *Callitriche* genus.	*Callitriche obtusangula*
		*Callitriche platycarpa*
Broad leaved macrophytes	Any submerged macrophytes deemed to have broad leaves.	*Berula erecta*
		*Helosciadium nodiflorum*
Filamentous green algae	Any submerged filamentous green algae.	*Cladophora glomerata*
Tape grass	Submerged macrophytes with long, flattened leaves splitting at the base of the plant.	*Schoenoplectus lacustris*
		*Sparganium emersum*

Examples of common species found within each group are provided. The groups were selected as they encompass the typical dominant taxa found in each river (e.g. [78]).

### 2.4 Macroinvertebrates

Macroinvertebrate samples were collected at the same times as for physical habitat data at eight transects in both restored and control sites per sampling date (i.e. at every second transect, at 20 m intervals) using a standard 3-minute kick sample (250 mm^2^ net, 1 mm mesh), or sweep sample from a boat if depth restricted wading [79]. This was followed by a 1-minute hand search for macroinvertebrates not likely to be found in the kick sample, such as those residing under larger substrate or woody material [79]. To ensure standardised sampling, the technique (i.e. kick or sweep sample) used during the first sampling period was maintained throughout the study. Over the 3-minute kick sampling period, substrates and all habitat types (e.g. macrophytes) were sampled in estimated proportion to their occurrence in the respective transect to provide appropriate representation of invertebrates from each microhabitat. Following collection, samples were placed in 1.2 L sampling pots and fixed with 70% methylated spirit.

Samples were sorted and macroinvertebrates identified within one month of collection. Each sample was poured through a 30 μm sieve and washed lightly with tap water to remove methylated spirit, before being placed into a tray with water and evenly distributed throughout. Samples were sub-sampled by dividing the area of the tray in half and sorting a random half by hand, collecting any macroinvertebrates present. These were identified by a single Freshwater Biological Association trained practitioner to the family level (Oligochaeta was classified as such) using a compound microscope (Motic ST-30) and identification guide [80]. Following identification, the abundance, taxon richness, EPTA, EPTN, LIFE [60] and PSI [59] was calculated (see S2 Appendix for description and calculation). These metrics were specifically selected as they provide information on community responses to changes in physical habitat (e.g. LIFE and PSI provide information on flow and silt pressures, with lower values suggesting a more lentic, silt-tolerant community, respectively [59, 60]) or encompass an important component of chalk stream ecology and food chains (e.g. EPT taxa [52, 81]). No permits were required to sample macroinvertebrates.

### 2.5 Statistical analysis

To evaluate the influence of time since restoration on physical habitat and ecological responses, the analysis was divided into two periods: immediate (0–1 years) and short-term (1–2 years) post restoration. Separate statistical models were created for EL and HS as a direct comparison between the two sites was not the focus of this study. Each dominant substrate type was summed across each transect and VCSV was square-root transformed. Analyses were carried out in R Studio [82] and the packages Lme4 [83], LSmeans [84], performance [85], ggplot2 [86], patchwork [87], nparLD [88], nparcomp [89] and MuMIn [90].

Physical habitat (excluding substrate cover) and macroinvertebrate metrics were analysed using generalised linear mixed models (GLMM; macroinvertebrate abundance [HS] and taxon richness [HS and EL]) with a Poisson error distribution and log-link function and linear mixed models (LMMs; all other physical and macroinvertebrate metrics). Models encompassed the terms ‘sampling period’ (pre-restoration, 0–1, 1–2 years post-restoration), ‘location’ (restored or control site) and the ‘interaction between sampling period x location’. ‘Sampling date’ and ‘sampling point/transect’ were considered random effects to account for pseudoreplication. Random effect structures were automatically assigned in Lme4 and were nested in sampling period and site, respectively. All metrics were categorical. In these models, significant sampling period x location interactions suggest an effect of the restoration. The significance of each variable was assessed using likelihood ratio tests by comparing full models with one with the focal terms removed. For all LMMs and GLMMs, multicollinearity and model assumptions were checked using variance inflation factor and QQ and fitted vs residual plots, respectively. Random effect variance and R² values were recorded and are available in S3 Appendix. TukeyHSD pairwise comparisons were performed post-hoc when sampling period or sampling period x location interactions were significant.

For macrophyte and substrate metrics, diagnostic plots (e.g. QQ and fitted vs residual plots) suggested model assumptions (e.g. normality of residuals) were not met by mixed models. Therefore, a non-parametric rank-based repeated measures approach was conducted using ‘nparLD’ [88]. This approach does not require any distribution assumptions, is considered robust to small and variable samples sizes and outliers, and provides an ‘ANOVA-type’ statistic (ATS [88]). Where multiple samples were taken within a sampling period, a single value for each sampling point/transect was calculated by taking the mean across each period, a necessity of the statistical test. Models were created with the same terms as for the parametric analysis, although ‘sampling date’ was not included due to averaging across each sampling period. Where sampling period or sampling period x location interactions were significant for macrophyte and substrate metrics, post-hoc pairwise comparisons were conducted in ‘nparcomp’ and using the function ‘mctp.rm’ with TukeyHSD corrections [89]. For significant interactions, pairwise comparisons were used to test differences between sampling periods on separate models for restored and control reaches.

## 3. Results

### 3.1. Physical habitat

**Fig 3:**
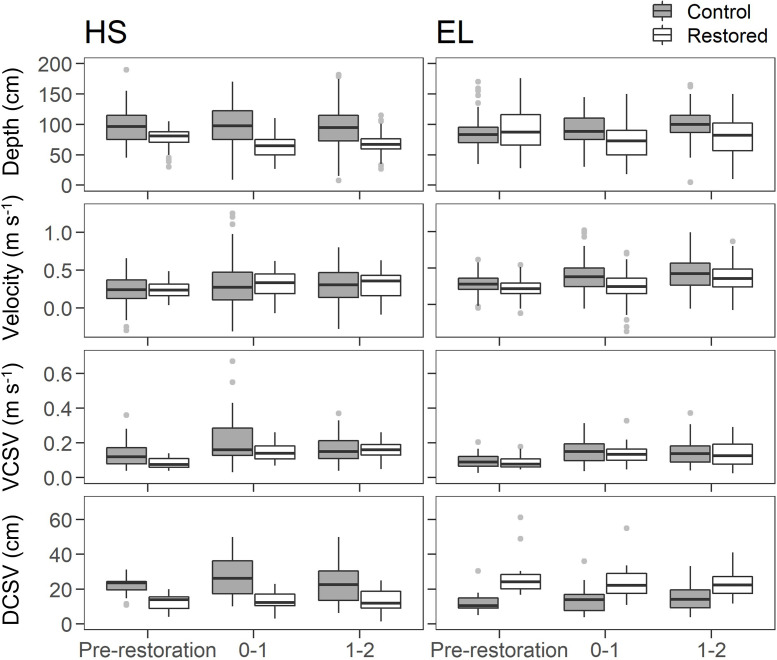
Changes in physical habitat metrics in HS and EL restored and control sites prior to and 0–1 and 1–2 years post gravel augmentation. Black bars and boxes indicate median and 25^th^ and 75^th^ percentiles, respectively. Whiskers represent minimum and maximum values excluding outliers. Dots show outliers (values > 1.5 x the interquartile range).

**Fig 4:**
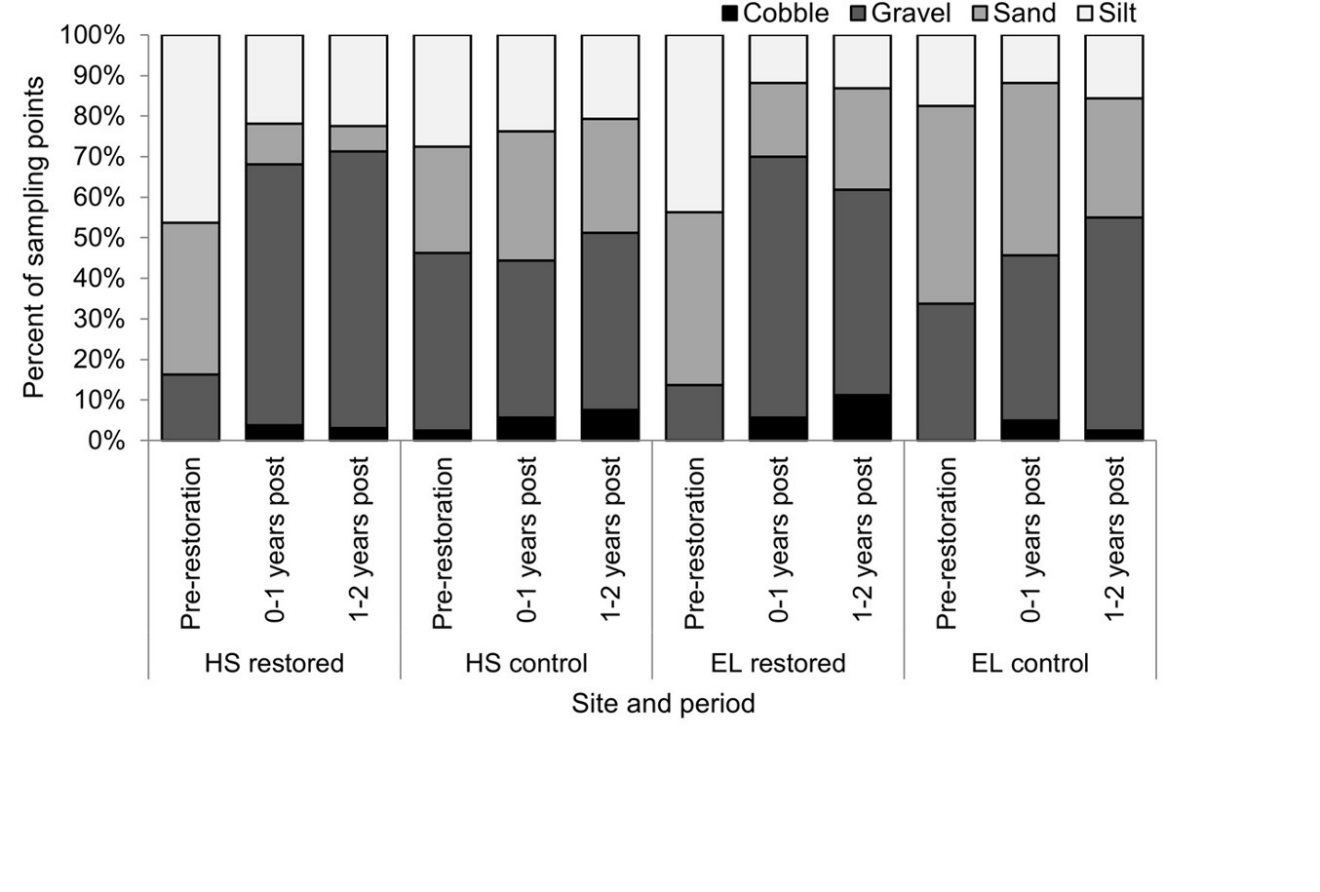
Substrate composition in restored and control reaches in HS and EL prior to and 0–1 and 1–2 years post gravel augmentation.

Interactions between sampling period and location were observed for depth, VCSV and the cover of sand and gravel substrates in HS (Figs 3 and 4 and Table 2). Depth and sand cover was lower, and gravel cover higher during both post-restoration periods in the restored reach compared to pre-restoration (see S4 Appendix for post-hoc results). Compared to pre-restoration, VCSV was higher 1–2 years post-restoration in the restored reach. No difference in metrics between periods was observed for the control site.

**Table 2:** Results of generalised (GLMM) and linear mixed models (LMM) and non-parametric repeated measures models (NP) used to assess the responses of physical habitat and ecological metrics to gravel augmentation.

Site	Type	Term	Test	Sampling period x Location		Location		Sampling period	
				*X2*/ATS	p	*X2*/ATS	p	*X2*/ATS	p
HS	Physical	Depth	LMM	28.142	**< 0.001**	63.439	**< 0.001**	7.260	**< 0.05**
		Velocity	LMM	0.013	0.994	0.087	0.768	4.901	0.086
		DCSV	LMM	5.713	0.057	21.219	**< 0.001**	2.097	0.351
		VCSV	LMM	6.939	**< 0.05**	4.106	**< 0.05**	7.698	**< 0.05**
		Silt	NP	1.507	0.223	0.763	0.383	3.439	**< 0.05**
		Sand	NP	12.999	**< 0.001**	7.030	**< 0.01**	5.429	**< 0.01**
		Gravel	NP	26.222	**< 0.001**	2.985	0.084	18.349	**< 0.001**
		Cobble	NP	0.290	0.674	0.572	0.450	5.055	**< 0.05**
	Macrophytes	TMC	NP	2.586	0.077	11.682	**< 0.001**	10.770	**< 0.001**
		FGA	NP	4.490	**< 0.05**	0.674	0.412	6.070	**< 0.01**
		Water crowfoot	NP	0.125	0.833	62.135	**< 0.001**	6.507	**< 0.01**
		BL macrophyte	NP	0.505	0.593	3.085	0.079	0.444	0.630
		Tape grass	NP	0.433	0.640	31.397	**< 0.001**	4.878	**< 0.01**
		Water starwort	NP	0.410	0.658	3.258	0.071	0.431	0.645
	Macroinvertebrates	Abundance	GLMM	158.070	**< 0.001**	2.117	0.146	4.861	0.088
		Taxon richness	GLMM	13.994	**< 0.001**	1.547	0.214	4.682	0.096
		EPTA	LMM	3.706	0.157	21.854	**< 0.001**	3.120	0.210
		EPTN	LMM	6.542	**< 0.05**	5.208	**< 0.05**	3.758	0.153
		PSI	LMM	13.803	**< 0.01**	5.166	**< 0.05**	8.722	**< 0.05**
		LIFE	LMM	6.259	**< 0.05**	4.803	**< 0.05**	1.034	0.596
EL	Physical	Depth	LMM	51.031	**< 0.001**	19.547	**< 0.001**	7.088	**< 0.05**
		Velocity	LMM	7.377	**< 0.05**	16.214	**< 0.001**	5.871	0.053
		DCSV	LMM	3.301	0.192	28.812	**< 0.001**	0.871	0.647
		VCSV	LMM	0.145	0.930	0.566	0.452	2.450	0.294
		Silt	NP	3.987	**< 0.05**	2.590	0.108	7.139	**< 0.001**
		Sand	NP	6.226	**< 0.01**	7.114	**< 0.01**	8.284	**< 0.001**
		Gravel	NP	9.908	**< 0.001**	0.136	0.712	18.402	**< 0.001**
		Cobble	NP	3.852	**< 0.05**	6.643	**< 0.01**	13.931	**< 0.001**
	Macrophytes	TMC	NP	1.077	0.329	2.642	0.104	13.876	**< 0.001**
		FGA	NP	1.900	0.157	1.967	0.161	10.657	**< 0.001**
		Water crowfoot	NP	3.297	0.053	0.244	0.621	15.012	**< 0.001**
		BL macrophyte	NP	3.742	**< 0.05**	1.000	0.317	26.202	**< 0.001**
		Tape grass	NP	3.625	**< 0.05**	0.300	0.584	0.697	0.478
		Water starwort	NP	0.964	0.376	0.915	0.339	0.532	0.574
	Macroinvertebrates	Abundance	LMM	2.628	0.269	0.000	0.991	1.890	0.389
		Taxon richness	GLMM	7.570	**< 0.05**	3.720	0.054	9.452	**< 0.01**
		EPTA	LMM	5.780	0.056	0.392	0.532	0.912	0.634
		EPTN	LMM	3.145	0.208	0.906	0.341	8.577	**< 0.05**
		PSI	LMM	7.862	**< 0.05**	3.103	0.078	8.264	**< 0.05**
		LIFE	LMM	1.522	0.467	3.429	0.064	6.797	**< 0.05**

Significant p-values are highlighted in bold. Location = control or restored reach, Sampling period = pre-restoration, 0–1 or 1–2 years post-restoration (0–1 and 1–2 years only for fish). ATS = ‘ANOVA-type statistic’; TMC = total macrophyte cover, FGA = filamentous green algae; BL = broad leaved.

In EL, interactions between sampling period and location were observed for depth, velocity, and cover of silt, sand, gravel and cobble. In the restored reach, silt cover was lower, and gravel and cobble cover higher, during both post-restoration periods compared to pre-restoration. Gravel cover was higher 0–1 years compared to 1–2 years post-restoration. Compared to pre-restoration, sand cover and depth was lower 0–1 years but not 1–2 years post-restoration in the restored reach. Velocity in the restored reach was lower than the control 0–1 years post-restoration. Aside from higher gravel cover 1–2 years post-restoration than the pre-restoration period, there was no change in physical habitat metrics over time in the control site.

### 3.2. Macrophytes

**Fig 5:**
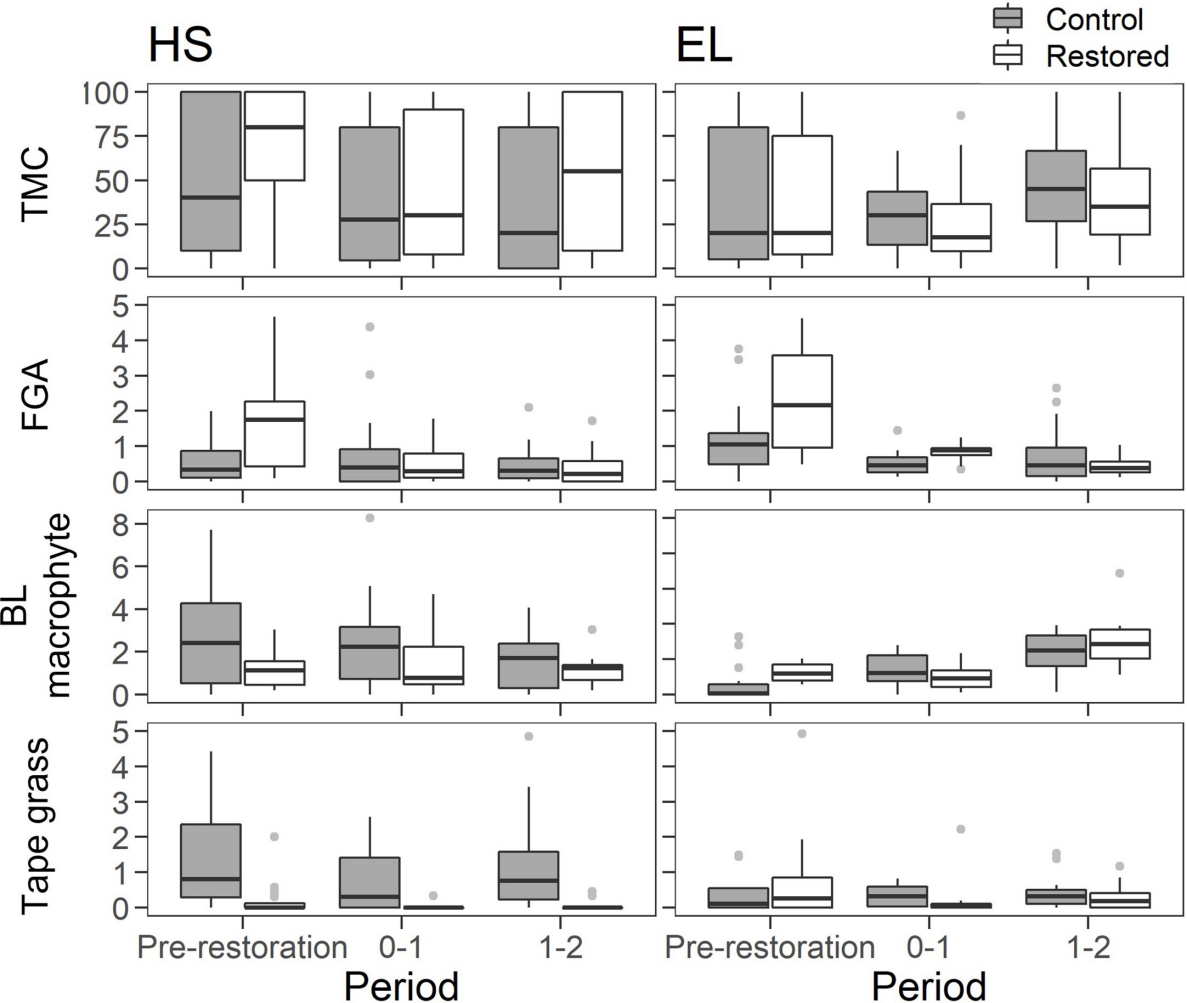
The changes in total macrophyte cover (TMC) and filamentous green algae (FGA), broad leaved (BL) macrophyte and tape grass (m^2^) in HS and EL restored and control sites prior to and 0–1 and 1–2 years post-restoration with gravel augmentation. Black bars and boxes indicate median and 25^th^ and 75^th^ percentiles, respectively. Whiskers represent minimum and maximum values excluding outliers. Dots show outliers (values > 1.5 x the interquartile range).

Total macrophyte cover in HS and EL was influenced by sampling period, and in the case of HS also by location (Table 2 and Fig 5). There was no interaction between sampling period and location (Table 2). Total macrophyte cover was higher prior to restoration compared to 0–1 and 1–2 years post-restoration in HS, and higher 1–2 years post-restoration compared to pre- and 0–1 years post-restoration in EL (S4 Appendix). In HS, total macrophyte cover was higher overall in the restored compared to the control reach.

In HS, an interaction between sampling period and location for filamentous green algae indicated lower cover in the restored reach during both post-restoration periods compared to pre-restoration, while there was no difference over time for the control reach. In EL, an interaction between sampling period and location was observed for broad leaved macrophytes and tape grass (Table 2). However, this was marginal and post-hoc comparisons indicated the overall responses of these groups did not differ between the restored and control reach.

### 3.3. Macroinvertebrates

In total, 11,963 and 18,835 individual macroinvertebrates representing 66 and 63 families were sampled in HS and EL, respectively. Prior to restoration, dominant families were Gammaridae (mean abundance per sample ± SD = 22.3 ± 18.4), Ephemeridae (8.6 ± 6.0) and Aphelocheiridae (7.6 ± 9.6) in HS, and Gammaridae (28.5 ± 31.5), Ephemerellidae (28.5 ± 18.3) and Bithyniidae (21.5 ± 16.5) in EL. One year after the initial survey in HS, Gammaridae (51.4 ± 30.1) and Ephemeridae (25.6 ± 15.0) remained the most abundant families, but Valvatidae (19.4 ± 17.3) exhibited increasing numbers. Likewise, in EL, Gammaridae (113.1 ± 47.8) and Ephemerellidae (90.3 ± 55.7) remained most abundant, but Baetidae (31.4 ± 31.4) became increasingly more frequent. The most abundant taxa found two years following the initial surveys remained similar at the end of the study period, although the values did vary. Indeed, Gammaridae (65.1 ± 22.9) and Valvatidae (17.8 ± 9.4) remained abundant, but Baetidae (38.9 ± 24.7) replaced Ephemeridae as the second most abundant taxa in HS. Gammaridae (40.5 ± 34.1), Ephemerellidae (36.0 ± 25.8) and Baetidae (38.9 ± 20.8) were most abundant in EL.

**Fig 6:**
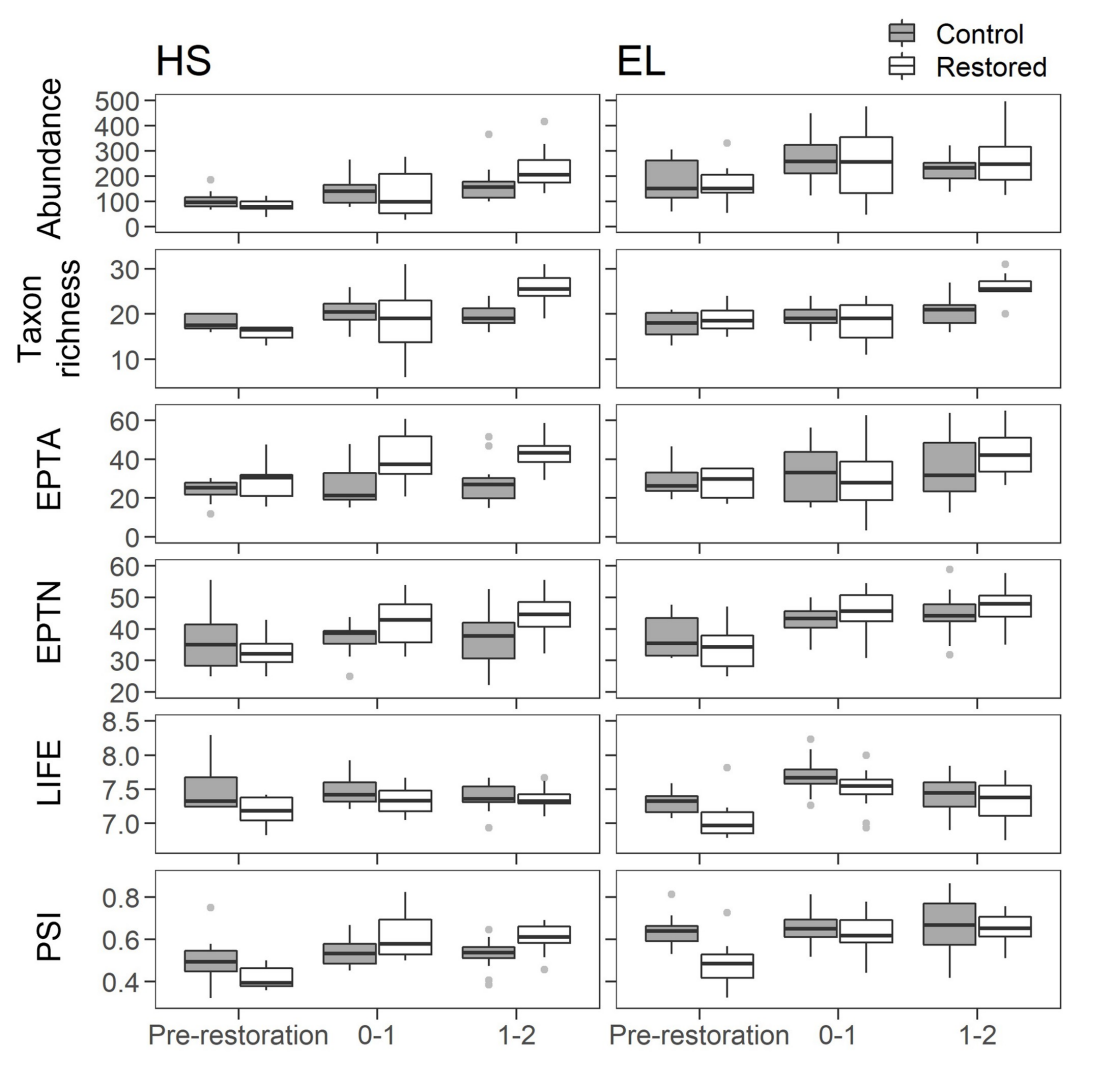
The changes in macroinvertebrate metrics in HS and EL restored and control sites prior to and 0–1 and 1–2 years post-restoration with gravel augmentation. Black bars and boxes indicate median and 25^th^ and 75^th^ percentiles, respectively. Whiskers represent minimum and maximum values excluding outliers. Dots show outliers (values > 1.5 x the interquartile range).

In HS and EL, interactions between sampling period and location were observed for taxon richness and PSI (Table 2 and Fig 6). Compared to pre-restoration values, the restored sites had a higher PSI in both post-restoration periods and a higher taxon richness 1–2 years post-restoration (S4 Appendix). Taxon richness was also lower for both sites 0–1 years compared to 1–2 years post-restoration. In HS, an interaction between sampling period and location was also observed for abundance, LIFE and EPTN. Abundance in the restored reach was higher 1–2 years post-restoration compared to pre-restoration. Post-hoc comparisons did not indicate a change in LIFE or EPTN across periods in the restored or control reach, although LIFE was lower in the restored compared to the control reach prior to restoration. In both HS and EL, there was no change in macroinvertebrate metrics between periods in the control reach.

## 4. Discussion

Although gravel augmentation is commonly used to improve the physical and ecological condition of sediment depleted rivers [23], the impact on different ecological groups remains poorly understood. This study investigated the physical and ecological responses to gravel augmentation at two chalk stream restoration sites in southern England. Unsurprisingly, and in support of our first prediction, changes in physical habitat metrics were often immediate (e.g. increased gravel cover) and remained so for the duration of the study. However, other factors (e.g. velocity) showed little change. Likewise, and in support of our second prediction, the consequent ecological response could be considered mostly positive in light of typical restoration goals (e.g. to improve biodiversity), but also varied between sites and ecological groups. For example, while there was little change in macrophytes following restoration, clearer responses were observed for macroinvertebrates (e.g. enhanced dominance of silt-intolerant taxa). Furthermore, in support of our third prediction the ecological response was often influenced by time. For example, an increase in macroinvertebrate abundance (HS only) and taxon richness was only observed 1–2 years post-restoration.

When applied at the reach scale, as in this study, the addition of gravel to a degraded river represents a feature-based restoration approach intended to enhance localised physical habitat [24]. Such interventions are hoped to result in a desired outcome related to improvement in some measure of ecological condition (e.g. ecological status: Water Framework Directive [91]; biodiversity net gain: United Kingdom Environment Act [92]). In chalk streams, which tend to be highly engineered, managed and exhibit stable hydrogeomorphology, we predicted that changes to physical habitat would be rapid, and predominantly relate to a reduction in depth, and increased velocity, habitat heterogeneity and coarse substrate cover. This expectation was partially realised. At both sites, restored reaches became shallower (EL 0–1 years post-restoration only), more gravel-dominated, and velocity became more heterogeneous in HS (1–2 post-restoration only), possibly aided by a small number of felled trees. Conversely, mean velocity showed little change. Although unlikely, the lower velocity in the EL restored reach compared to the control 0–1 years post-restoration may have resulted from slight variation in flow between sampling restored and control reaches. Furthermore, although gravel cover increased following restoration, at EL cover was lower 1–2 compared to 0–1 years post-restoration, possibly because fine sediment input [62] confounded the influence of gravel addition the year following restoration. Continued fine sediment deposition might smother introduced coarse substrates and compromise the long-term success of such an approach [28, 93], illustrating the potential for the benefits of reach-scale interventions to be confounded by catchment-scale processes [94, 95]. This problem is especially relevant in chalk streams that typically exhibit stable flow regimes and limited stream power to redistribute fine sediments [56]. This emphasises the need to understand the underlying root causes of degradation and for restoration actions to take place over commensurate scales [94]. Unexpectedly, the proportion of gravel in the control site at EL increased over time and was higher 1–2 years post-restoration compared to pre-restoration, possibly a result of difference in flow across sampling periods. It would be of interest to also monitor natural changes in sediment grainsize and underlying causes of any fluctuations observed. Due to differences in the perception of river restoration of those involved in the United Kingdom context (e.g. key stakeholders can range from pro- to anti-restoration), and consequent willingness of riparian landowners to participate, holistic catchment-scale targets are more likely to be achieved if the planning process is based on pragmatic opportunism (undertaking restoration when possible, e.g. when stakeholder agreement and funding align) that embraces what may first appear to be a rather piecemeal approach. As such, reach-scale interventions that may represent feature based approaches when viewed in isolation, may also enable the reestablishment of more natural hydrogeomorphological processes over greater spatial and temporal scales [94], perhaps reversing processes of ecological degradation that occurred over centuries of river engineering.

Gravel augmentation was predicted to bring about desirable ecological change in line with the restoration and overarching catchment management aims (e.g. River Test and Itchen Restoration Strategy [62]). For the most part this prediction was realised, although responses varied considerably between ecological groups and sites. Indeed, macroinvertebrates responded strongly to restoration. At HS they became more abundant, and at both sites became more taxon rich and increasingly dominated by silt-intolerant taxa. This may have been due to an increase in suitable habitat within the interstitial spaces of the coarser substrate added [96, 97], and the redistribution of fine sediments. In contrast, the response of macrophytes to restoration was comparatively weak, despite translocation of *Ranunculus* spp. Only a reduction in the cover of filamentous green algae was observed at HS, potentially due to the redistribution of nutrients within fine sediment [98] or because Cladophora are mid to late successional species [99] and require a longer colonisation time than provided in this study. Responses to restoration can be complex and vary between systems and ecological groups due to a variety of interacting factors. For example, catchment size [100], watershed degradation [28], substrate mobility [44] and recolonisation potential (e.g. species pools [101]) can all influence recovery, whilst differences in organism group (e.g. benthic versus open water groups), species (e.g. salmonid versus lamprey substrate requirements [29, 102]) and life-stage [93] habitat preferences can further complicate the prediction of outcomes. Gaining a deeper comprehension of the factors that contribute to variation in response is important for reducing uncertainty and advancing restoration practices. To achieve this, there is a need to promote the wider adoption of monitoring and its timely dissemination as a key part of the restoration process [103]. This includes the reporting of results that contradict study predictions or projects aims, which can provide valuable insights when implementing new projects.

The manipulation of river habitat through restoration can lead to localised ecological disturbance that may delay the realisation of desirable outcomes as communities recover [32, 104, 105]. For this reason, many have argued that the time-scales over which monitoring should occur must be sufficient to capture the response investigated (e.g. [46]); in many studies this is not the case [106]. We predicted that the ecological response may be limited or even negative (e.g. decline in abundance and taxon richness) when measured within one-year of restoration and that desirable outcomes (e.g. increased abundance and taxon richness) may take longer to accrue, i.e. between one and two years post-restoration. In our study, negative impacts were limited, with most metrics showing either positive responses (e.g. a shift towards more silt-intolerant macroinvertebrate taxa) or no change. As predicted, positive responses in several metrics (e.g. macroinvertebrate abundance [HS only] and taxon richness) became apparent only between one and two years after restoration, reflecting the time required for colonisation of the restored sites [107].

Despite an increase in river restoration over recent decades, robust and rigorous monitoring of effectiveness against stated aims remains relatively rare [108]. The lack of sufficient monitoring, independent of time-scale, is perhaps unsurprising considering the costs of doing so relative to the overall budget available for individual projects, particularly for those that are relatively small [109]. One approach to resolve this challenge is to develop collaborative and co-ordinated river restoration networks to share resources and strategically plan programmes in which representative (flag-ship) projects are selected for robust monitoring over appropriate spatial and temporal scales [110]. Likewise, best practice guidance for monitoring should be updated considering the technological advance that has occurred over recent times [111], including in genetics, filming, telemetry, remote sensing, and artificial intelligence. Doing so will advance the collection of appropriate evidence on which future decision making can be based, allowing lessons to be learnt from both successes and failures.

This study indicates that gravel augmentation can positively benefit degraded chalk stream habitat and ecology, at least over limited spatial and temporal scales. However, responses to such interventions can be variable due to a multitude of factors, including physical and chemical attributes of the site, the ecological group considered, and the time since restoration. Whilst not assessed in this study, the responses to gravel augmentation will likely vary across river types, emphasising the need for robust monitoring to facilitate the development of system-specific best practice. For example, in chalk streams and other low power groundwater dominated systems, augmented gravels may be less prone to redistribution and more susceptible to sedimentation compared with more powerful surface-fed systems [28, 44, 93]. This will likely reduce the long-term benefits of such projects and emphasises the need for gravel augmentation to be carried out alongside a range of interventions to reinvigorate natural processes (e.g. reduction of catchment-scale sediment inputs, recolonisation of gravel mobilising ecosystem engineers, addition of woody materials [52]). In addition to ensuring strategically selected projects are rigorously monitored using appropriate scientific techniques and field study design over appropriate spatial and temporal scales, there is also a need to adopt more holistic approaches that consider community level response, moving away from a single target species bias that typically focuses on fish [23]. The use of feature-based approaches, not in isolation, but as part of a spatially and temporally broader and integrated strategy to reinstate process-based river re-naturalisation is likely to yield considerable benefits in the long-term. Just as rivers were degraded through engineering practices over many centuries, their regeneration will take time and likely depend on opportunistic reach-scale projects apparently conducted in a piecemeal fashion in a resource limited environment. However, the effectiveness of such may be enhanced if applied within a strategic catchment scale management framework that recognises the false dichotomy between the relative values of feature versus process-based restoration.

## Supporting information

S1 Appendix Schematic and example calculation of the cover of macrophyte groups across each transect. Thick lines show river boundary. Hashed area shows the 1 m area across the transect which was assessed. Boxes show the five percent total macrophyte cover (TMC) estimates made across each transect. Letters and coloured areas show different macrophyte types.
(TIF)

S2 Appendix Details of the macroinvertebrate metrics used in a study to investigate ecological response to chalk stream gravel augmentation and how they were calculated. (DOCX)

S3 Appendix Random effect variances, standard deviations (SD) and marginal and conditional R^2^ values for full linear and generalised linear mixed models. (DOCX)

S4 Appendix Results of Tukey pairwise comparison post-hoc tests following significant before-after x control-impact interactions (A and C) and before-after (B) terms. Significant comparisons are boldened. FGA = filamentous green algae; TMC = total macrophyte cover.
(DOCX)
