# Supplementary figures and images for: Chalk stream restoration: Physical and ecological responses to gravel augmentation
Source: PLoS One. 2024 Nov 20;19(11):e0313876. doi: 10.1371/journal.pone.0313876 (PMC11578525; doi:10.1371/journal.pone.0313876)

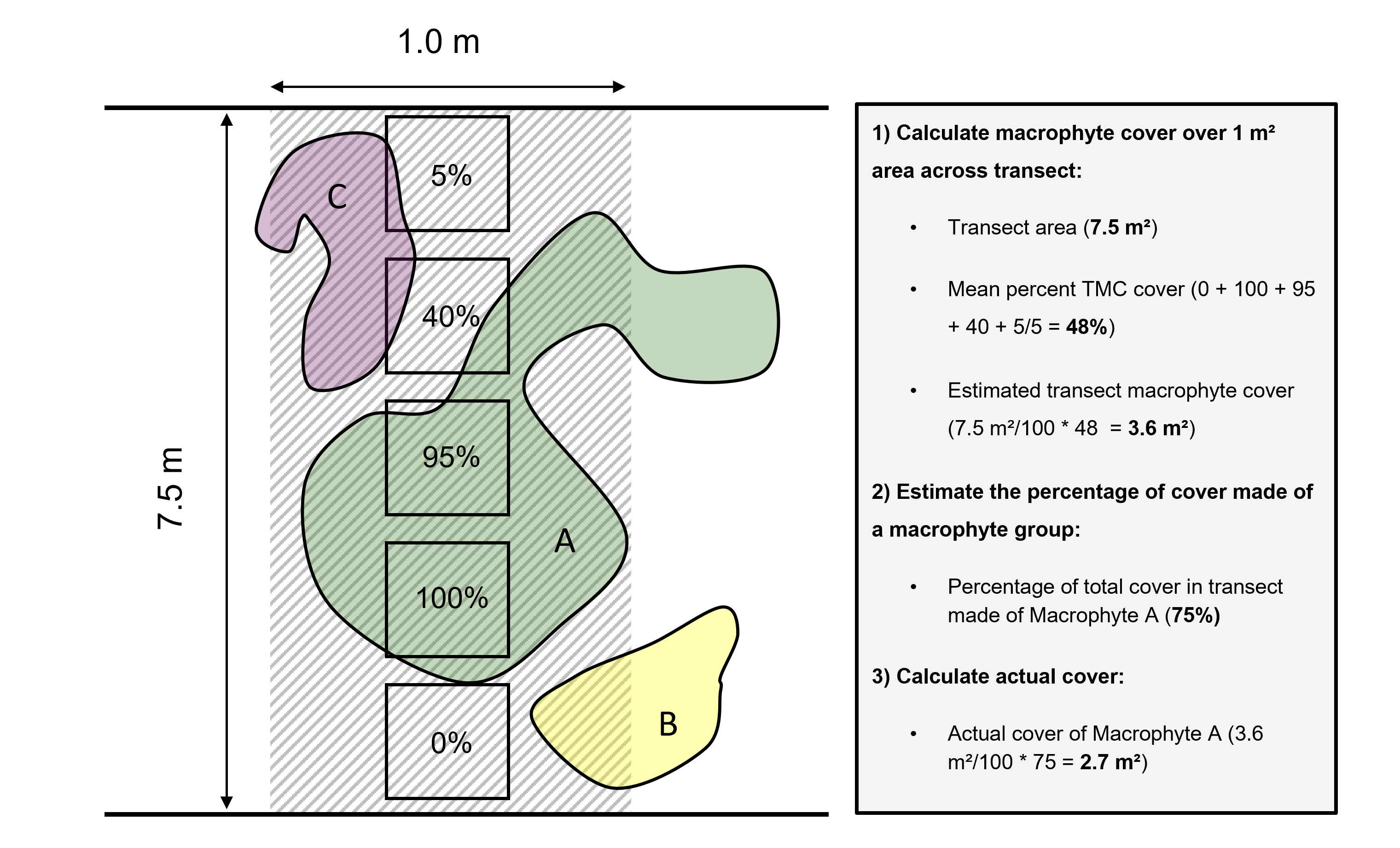

Supplement: S1 Appendix — Thick lines show river boundary. Hashed area shows the 1 m area across the transect which was assessed. Boxes show the five percent total macrophyte cover (TMC) estimates made across each transect. Letters and coloured areas show different macrophyte types. (TIF) [file pone.0313876.s001.tif]
