# Supplementary material for: Chalk stream restoration: Physical and ecological responses to gravel augmentation
Source: PLoS One. 2024 Nov 20;19(11):e0313876. doi: 10.1371/journal.pone.0313876 (PMC11578525; doi:10.1371/journal.pone.0313876)
Supplement: S2 Appendix — (DOCX) [file pone.0313876.s002.docx]

| S2 Appendix: Details of the macroinvertebrate metrics used in a study to investigate ecological response to chalk stream gravel augmentation and how they were calculated. | |
| --- | --- |
| **Metric** | **Description** |
| Abundance | The total number of individuals within the sample. |
| Taxon richness | The total number of families within the sample. This metric is a measure of diversity. |
| EPTA | Ephemeroptera, Plecoptera, and Trichoptera (EPT) are commonly assessed in biological monitoring programmes due to their sensitivity to a range of environmental stressors [109]. Moreover, EPT taxa are important food sources for fish, especially salmonids [110]. EPTA assesses the percentage of the total abundance made of EPT, with lower values generally showing a more degraded system.  $EPTA= \frac{Abundance of EPT}{Abundance} x 100$ |
| EPTN | EPTN assesses the percentage of the taxon richness made of EPT, with lower values generally show a more degraded system.  $EPTN= \frac{Number of EPT taxa}{Taxon richness} x 100$ |
| LIFE | Used to assess the impact of flow velocity on a system. Each family is given a flow tolerance score based on their abundance within the sample (<10, <100, <1000, >1000). LIFE is calculated as the sum of all LIFE scores within the sample divided by the number of LIFE rated families within the sample. Lower scores show a community comprised of more flow-intolerant taxa and a lower flow velocity in the system. Scores and calculation from [76]. |
| PSI | Used to assess the impact of silt on a system. Each family is given a silt tolerance score based on their abundance within the sample (<10, <100, <1000, >1000): A: highly sensitive, B: moderately sensitive, C: moderately insensitive, D: highly insensitive. PSI is calculated as:  $PSI= \frac{\sum(Scores for groups A \& B)}{\sum(Scores for groups A, B, C \& D)}$  A score between 0 and 1 is created, with lower scores showing a community comprised of more silt-tolerant taxa and a higher impact of silt on a system. Family groupings and calculations from [77]. |
