# Supplementary material for: Chalk stream restoration: Physical and ecological responses to gravel augmentation
Source: PLoS One. 2024 Nov 20;19(11):e0313876. doi: 10.1371/journal.pone.0313876 (PMC11578525; doi:10.1371/journal.pone.0313876)
Supplement: S3 Appendix — (DOCX) [file pone.0313876.s003.docx]

| **Site** | **Term** | **Location** | | **Month** | | **Marginal R^2^** | **Conditional R^2^** |
| --- | --- | --- | --- | --- | --- | --- | --- |
|  |  | **Variance** | **SD** | **Variance** | **SD** |  |  |
| **East Lodge** | Depth | 335.30 | 18.31 | 2.60 | 1.61 | 0.12 | 0.54 |
|  | Velocity | 0.01 | 0.12 | 0.00 | 0.04 | 0.12 | 0.48 |
|  | DCSV | 9.48 | 3.08 | 0.00 | 0.00 | 0.33 | 0.43 |
|  | VCSV | 0.00 | 0.04 | 0.00 | 0.03 | 0.08 | 0.39 |
|  | Abundance | 459.40 | 21.43 | 1838.10 | 42.87 | 0.13 | 0.36 |
|  | Taxon richness | 0.00 | 0.00 | 0.00 | 0.00 | 0.27 | 0.27 |
|  | EPTA | 1.72 | 1.31 | 93.92 | 9.69 | 0.13 | 0.58 |
|  | EPTN | 0.00 | 0.00 | 0.76 | 0.87 | 0.30 | 0.31 |
|  | PSI | 0.00 | 0.02 | 0.00 | 0.00 | 0.23 | 0.26 |
|  | LIFE | 0.02 | 0.13 | 0.01 | 0.07 | 0.31 | 0.52 |
| **Home Stream** | Depth | 344.80 | 18.57 | 0.00 | 0.00 | 0.24 | 0.63 |
|  | Velocity | 0.02 | 0.15 | 0.00 | 0.02 | 0.02 | 0.49 |
|  | DCSV | 22.65 | 4.76 | 2.11 | 1.45 | 0.35 | 0.57 |
|  | VCSV | 0.00 | 0.05 | 0.00 | 0.01 | 0.17 | 0.43 |
|  | Abundance | 0.02 | 0.15 | 0.05 | 0.22 | 0.55 | 0.96 |
|  | Taxon richness | 0.00 | 0.00 | 0.01 | 0.07 | 0.28 | 0.35 |
|  | EPTA | 0.00 | 0.00 | 7.37 | 2.72 | 0.38 | 0.43 |
|  | EPTN | 0.00 | 0.00 | 0.86 | 0.93 | 0.20 | 0.21 |
|  | PSI | 0.00 | 0.00 | 0.00 | 0.01 | 0.37 | 0.38 |
|  | LIFE | 0.00 | 0.06 | 0.00 | 0.00 | 0.16 | 0.23 |

S3 Appendix. Random effect variances, standard deviations (SD) and marginal and conditional R^2^ values for full linear and generalised linear mixed models.
