# Supplementary material for: Chalk stream restoration: Physical and ecological responses to gravel augmentation
Source: PLoS One. 2024 Nov 20;19(11):e0313876. doi: 10.1371/journal.pone.0313876 (PMC11578525; doi:10.1371/journal.pone.0313876)
Supplement: S4 Appendix — Results of Tukey pairwise comparison post-hoc tests following significant before-after x control-impact interactions (A and C) and before-after (B) terms. Significant comparisons are boldened. FGA = filamentous green algae; TMC = total macrophyte cover. (DOCX) [file pone.0313876.s004.docx]

| S4 Appendix. Results of Tukey pairwise comparison post-hoc tests following significant before-after x control-impact interactions (A and C) and before-after (B) terms. Significant comparisons are boldened. FGA = filamentous green algae; TMC = total macrophyte cover. | | | | | | | | | | | | | | | | | | | | | | | |
| --- | --- | --- | --- | --- | --- | --- | --- | --- | --- | --- | --- | --- | --- | --- | --- | --- | --- | --- | --- | --- | --- | --- | --- |
|  |  | | | | | | | | | | | | | |  | | | | | | | | |
| A |  | | | | | | | | | | | | | |  | | | | | | | | |
| Contrast | HS | | | | | | | | | | | | | | EL | | | | | | | | |
|  | Depth |  | VCSV |  | Abundance | | Taxon richness | | EPTN |  | LIFE |  | PSI |  | Depth |  | Velocity | | Taxon richness |  | PSI |  |  |
|  | t | p | t | p | z | p | z | p | t | p | t | p | t | p | t | p | t | p | z | p | t | p |  |
| pre-control - pre-restored | 4.00 | **< 0.001** | 2.39 | 0.17 | 2.88 | **< 0.05** | 1.10 | 0.88 | 1.07 | 0.89 | 3.13 | **< 0.01** | 2.29 | 0.21 | -1.21 | 0.83 | 1.79 | 0.47 | -0.64 | 0.99 | 3.28 | **< 0.05** |  |
| 0-1 control - 0-1 restored | 9.80 | **< 0.001** | 2.52 | 0.13 | 0.75 | 0.98 | 1.17 | 0.85 | -1.82 | 0.46 | 1.51 | 0.66 | -2.70 | 0.09 | 5.43 | **< 0.001** | 4.89 | **< 0.001** | 0.82 | 0.96 | 0.53 | 0.99 |  |
| 1-2 control - 1-2 restored | 7.60 | **< 0.001** | 0.23 | 1.00 | -4.18 | **< 0.001** | -3.59 | **< 0.05** | -2.71 | 0.09 | 0.34 | 1.00 | -2.81 | 0.07 | 5.35 | **< 0.001** | 2.86 | 0.05 | -3.18 | 0.02 | 0.14 | 1.00 |  |
| 0-1 control - pre-control | 1.51 | 0.67 | 2.30 | 0.23 | 0.76 | 0.97 | 0.86 | 0.96 | 0.17 | 1.00 | -0.49 | 1.00 | 0.65 | 0.99 | 1.20 | 0.83 | 1.38 | 0.74 | 0.93 | 0.94 | 0.19 | 1.00 |  |
| 1-2 control - pre-control | 0.10 | 1.00 | 1.00 | 0.91 | 1.58 | 0.61 | 0.67 | 0.98 | 0.09 | 1.00 | -1.31 | 0.77 | 0.47 | 1.00 | 3.14 | 0.05 | 2.16 | 0.31 | 1.50 | 0.66 | 0.45 | 1.00 |  |
| 0-1 control - 1-2 control | 1.72 | 0.56 | 1.59 | 0.61 | -1.01 | 0.91 | 0.23 | 1.00 | 0.09 | 1.00 | 1.01 | 0.90 | 0.21 | 1.00 | -2.37 | 0.21 | -0.96 | 0.92 | -0.71 | 0.98 | -0.31 | 1.00 |  |
| 0-1 restored - pre-restored | -6.00 | **< 0.01** | 2.65 | 0.12 | 1.50 | 0.67 | 1.12 | 0.88 | 2.14 | 0.29 | 1.96 | 0.43 | 4.28 | **< 0.01** | -4.44 | **< 0.01** | 0.39 | 1.00 | -0.47 | 1.00 | 3.62 | **< 0.05** |  |
| 1-2 restored - pre-restored | -4.24 | **< 0.05** | 3.23 | **< 0.05** | 3.77 | **< 0.01** | 3.66 | **< 0.01** | 2.59 | 0.12 | 2.19 | 0.33 | 4.18 | **< 0.01** | -2.44 | 0.19 | 1.90 | 0.44 | 3.28 | **< 0.05** | 4.21 | **< 0.05** |  |
| 0-1 restored - 1-2 restored | -2.17 | 0.34 | -0.71 | 0.98 | -2.79 | 0.06 | -3.23 | **< 0.05** | -0.56 | 0.99 | -0.29 | 1.00 | 0.13 | 1.00 | -2.45 | 0.19 | -1.86 | 0.47 | -4.68 | **< 0.001** | -0.72 | 0.97 |  |

| B |  | | | | | | | | | |  | | | | | | | | | |
| --- | --- | --- | --- | --- | --- | --- | --- | --- | --- | --- | --- | --- | --- | --- | --- | --- | --- | --- | --- | --- |
| Contrast | HS | | | | | | | | | | EL | | | | | | | | | |
|  | Silt |  | Cobble | | TMC |  | Water crowfoot | | Tape grass | | TMC |  | FGA |  | Water crowfoot | | EPTN |  | LIFE |  |
|  | ATS | p | ATS | p | ATS | p | ATS | p | ATS | p | ATS | p | ATS | p | ATS | p | t | p | t | p |
| Pre-restored - 0-1 post-restoration | -1.78 | 0.19 | 3.07 | **< 0.05** | -4.44 | **< 0.001** | -3.12 | **< 0.01** | -3.35 | **< 0.01** | -1.54 | 0.27 | -2.81 | **< 0.05** | 1.45 | 0.32 | 2.48 | 0.07 | 2.40 | 0.08 |
| Pre-restored - 1-2 post-restoration | -2.10 | 0.10 | 3.48 | **< 0.01** | -2.83 | **< 0.05** | -2.12 | 0.10 | -1.48 | 0.32 | 2.94 | **< 0.05** | -2.83 | **< 0.05** | 5.13 | **< 0.001** | 2.94 | **< 0.05** | 1.02 | 0.58 |
| 0-1 - 1-2 years post-restoration | -0.34 | 0.94 | 0.27 | 0.96 | 1.59 | 0.25 | 1.82 | 0.18 | 1.58 | 0.27 | 7.33 | **< 0.001** | -0.92 | 0.62 | 4.45 | **< 0.001** | -0.55 | 0.85 | 1.69 | 0.25 |

| C |  | | | | | |  | | | | | | | | | | | |
| --- | --- | --- | --- | --- | --- | --- | --- | --- | --- | --- | --- | --- | --- | --- | --- | --- | --- | --- |
| Contrast | HS | | | | | | EL | | | | | | | | | | | |
|  | Sand |  | Gravel | | FGA |  | Silt |  | Sand |  | Gravel |  | Cobble | | BL macrophyte | | Tape grass | |
|  | ATS | p | ATS | p | ATS | p | ATS | p | ATS | p | ATS | p | ATS | p | ATS | p | ATS | p |
| 0-1 restored - pre-restored | -4.54 | **< 0.01** | 5.78 | **< 0.001** | -3.11 | **< 0.05** | -4.20 | **< 0.01** | -3.95 | **< 0.01** | 5.47 | **< 0.001** | 3.47 | **< 0.01** | -1.01 | 0.59 | -1.38 | 0.39 |
| 1-2 restored - pre-restored | -4.38 | **< 0.01** | 5.46 | **< 0.001** | -4.02 | **< 0.01** | -3.80 | **< 0.01** | -2.36 | 0.08 | 3.83 | **< 0.01** | 4.80 | **< 0.001** | 5.12 | **< 0.01** | -0.67 | 0.78 |
| 0-1 restored - 1-2 restored | -0.55 | 0.85 | 0.33 | 0.94 | -0.82 | 0.70 | 0.32 | 0.95 | 2.18 | 0.11 | -3.67 | **< 0.01** | 2.08 | 0.13 | 5.04 | **< 0.01** | 1.28 | 0.44 |
| 0-1 control - pre-control | 0.98 | 0.59 | -0.46 | 0.88 | 0.26 | 0.96 | -0.71 | 0.76 | -0.36 | 0.93 | 0.67 | 0.77 | 2.22 | 0.10 | 2.32 | 0.08 | 1.54 | 0.30 |
| 1-2 control - pre-control | 0.55 | 0.85 | 0.15 | 0.99 | -0.69 | 0.77 | -0.20 | 0.98 | -2.25 | 0.09 | 2.90 | **< 0.05** | 2.19 | 0.10 | 5.11 | **< 0.001** | 1.76 | 0.21 |
| 0-1 control - 1-2 control | -0.71 | 0.76 | 0.84 | 0.65 | -0.82 | 0.70 | 0.57 | 0.84 | -2.58 | 0.05 | 2.18 | 0.10 | -0.13 | 0.99 | 3.54 | **< 0.01** | 0.23 | 0.97 |
